# Development and validation of the message-framed exercise rehabilitation education program for post-PCI patients: a study protocol

**DOI:** 10.3389/fpubh.2026.1740229

**Published:** 2026-01-21

**Authors:** Yue Zhao, Yueying Jiang, Maidiniguli Maitikuerban, Meijuan Lan, Leiwen Tang

**Affiliations:** Department of Nursing, The Second Affiliated Hospital of Zhejiang University School of Medicine, Hangzhou, China

**Keywords:** coronary heart disease, exercise rehabilitation adherence, message framing, post-PCI patients, study protocol

## Abstract

**Background:**

Exercise rehabilitation is essential for improving the prognosis of patients after percutaneous coronary intervention. However, patients’ adherence to exercise rehabilitation is generally low. Existing health education interventions focus on the supply of message content, but generally ignore the impact of message expression on patients’ cognition and behavior, which limits the promotional effect of health education to a certain extent. The theory of message framing effects states that even a message that is essentially the same can have a differential impact on an individual’s behavioral decision-making through different ways of presenting it.

**Objective:**

This study aims to develop an educational program for exercise rehabilitation of post-PCI patients based on the effect of message framing, and to investigate the effects on exercise rehabilitation by integrating the gain-versus loss-framing and narrative versus non-narrative framing.

**Methods:**

In this study, we will first construct an exercise rehabilitation education program for post-PCI patients based on the message framing effect through qualitative interviews, evidence integration and Delphi expert consultation. A video-based presentation format will be developed simultaneously to deliver the intervention content. Subsequently, a 2 (gain vs. loss framing) × 2 (narrative vs. non-narrative framing) factorial randomized controlled design will be employed. Participants will be randomly assigned to four intervention groups to receive a three-week rehabilitation education intervention based on different message framing conditions. The primary outcome indicator is exercise adherence, and secondary outcomes include exercise intention, exercise fear, exercise self-efficacy and quality of life. Data will be collected at three time points: baseline, 1-month and 3-month post-intervention. Statistical analyses will be conducted using SPSS 25.0 and R 4.3.2 software, with linear mixed-effects models employed to assess the impact of different message framing conditions on primary and secondary outcomes across multiple time points.

**Discussion:**

This study will provide an innovative research method and a practical basis for exploring the optimal application of message framing effect in cardiac rehabilitation.

**Clinical trial registration:**

https://www.chictr.org.cn/index.html, Identifier ChiCTR2400087233.

## Introduction

Coronary atherosclerotic heart disease (CHD), as a major component of cardiovascular disease, poses a serious threat to patients’ lives and health. According to the *Report on Cardiovascular Health and Diseases in China 2023*, the number of cardiovascular patients with CHD in China is currently 11.39 million (1), and the prevalence and mortality rates continue to rise. Percutaneous Coronary Intervention (PCI) is a method of improving myocardial perfusion by unblocking narrowed or even occluded coronary artery lumen through cardiac catheterisation. With the advantages of being minimally invasive, fast recovery, and precise therapeutic effect, it has become one of the most important means of hemodialysis in coronary heart disease, and it can significantly reduce the acute stage mortality rate and improve life expectancy. However, PCI can only improve local stenosis, but cannot stop the progression of atherosclerosis (2, 3), and patients still face a high risk of angina recurrence, myocardial infarction, and even death after the procedure (4).

Cardiac rehabilitation (CR) has been recognized as a core measure for secondary prevention for patients after PCI. Exercise rehabilitation, as the core of CR, on one hand, can promote the establishment of coronary artery collateral circulation, effectively increase blood flow, improve myocardial metabolism and cardiac function, and reduce the rate of death (5, 6); on the other hand, it can help to control the risk factors of myocardial infarction, enhance the endurance of the patient’s activities, and improve the quality of life. Currently, exercise rehabilitation has been classified as the IA level recommended evidence by domestic and international guidelines and expert consensus (7–9). However, the participation rate of exercise rehabilitation in post-PCI patients is generally low (10, 11). Studies have shown that patients have insufficient knowledge of exercise rehabilitation (12), and most of them have a strong fear of exercise, which leads to poor exercise adherence and difficulty in obtaining rehabilitation benefits (13). Therefore, improving the knowledge of post-PCI patients about exercise rehabilitation and improving their adherence to exercise rehabilitation is a core problem that needs to be solved urgently.

Health message serves as a vital bridge between medical research findings and public health knowledge, attitudes, and behaviors (14). The accuracy, efficacy, and timeliness of its dissemination shape individuals’ understanding of health issues and, consequently, drive changes in health behaviors. Patients with cardiovascular disease often lack timely and effective message support at the time of hospital discharge, which greatly affects their motivation to participate in exercise rehabilitation (15). However, existing intervention studies on postoperative exercise adherence in PCI are mostly centered on health education, with messages delivered through educational brochures, bedside guidance, and short video pushes (16). Most of these studies are based on the perspective of healthcare supply, ignoring individual differences in patients’ cognitive level, emotional state, and message processing preferences, resulting in limited acceptance of the message, and patients’ low acceptance of the message on exercise rehabilitation, which eventually affects their willingness to participate in exercise and adherence to exercise (17).

The framing effect refers to the fact that different ways of describing a message based on the same problem result in generating different decision-making behaviors (18). Message framing examines how the presentation of a message influences the individual-message interaction, providing a new approach to studying how health communication drives behavior change (19). The message framing effect has been employed across various health domains, including disease detection (20, 21), vaccination (22, 23), smoking cessation (24), and physical exercise (25, 26). Prevalent message framing in health behavior research includes gain-loss framing, narrative framing, and temporal framing (27). The gain-loss framing is the most widely used type of goal framing, as it presents different forms of a message that is substantively the same by emphasizing the benefits to the individual of accepting a particular health behavior and the losses resulting from rejecting that behavior. However, the effectiveness of gain-loss framing in promoting improvement in individual health behaviors is controversial and has been less explored, especially in chronic disease conditions such as coronary heart disease, where there are fewer relevant studies with mixed results (28–30). In recent years, narrative framing has received increasing attention for its ability to enhance behavioral decision-making through the use of vivid, concrete stories that evoke emotional resonance and increase the acceptability, persuasiveness and memorability of the message (31). Research has shown that combining the gain-loss and the narrative framing can be more effective in enhancing the management of self-healthy behaviors (32, 33). Time framing influences individuals’ risk perceptions and behavioral intentions by altering their perceptions of time duration (34). Due to the repetitive and short-term nature of post-operative exercise rehabilitation after PCI, as well as increased patient risk awareness, time framing will not be included in the study. Instead, the study will focus on the integration of narrative and gain–loss framing.

## Objective

The study objectives are:

1. To investigate the characteristics of the message needs of exercise rehabilitation for post-PCI patients, and further construct an exercise rehabilitation education program for post-PCI patients based on the message framing effect through the summary of evidence and the Delphi method of expert correspondence.
2. To conduct a preliminary application of the exercise rehabilitation education program, exploring the effectiveness of differently framed exercise rehabilitation education.

## Methods

### Study design

**Figure 1:**
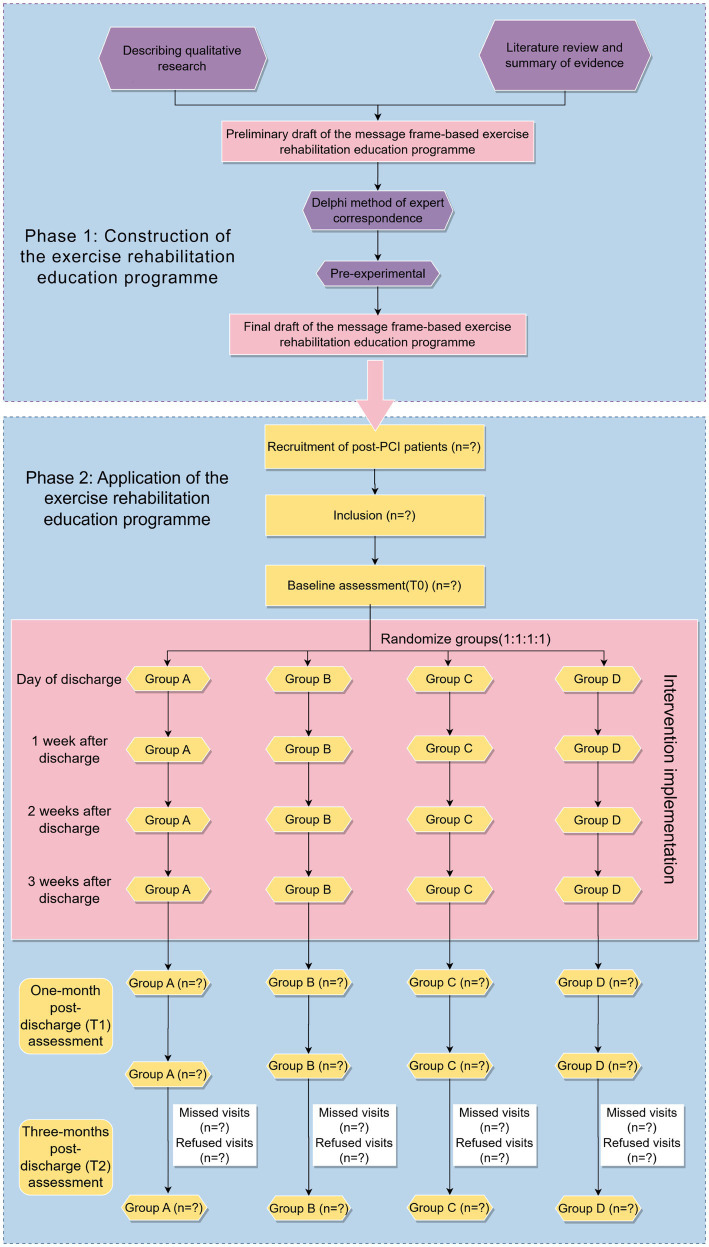
Study design and participant flow.

This is a two-phase study:

*Phase 1*: We will develop an exercise rehabilitation education program for post-PCI patients based on the message framing effect through qualitative needs assessment, evidence synthesis, and expert consensus.
*Phase 2*: We will conduct a prospective, parallel-controlled, single-blind randomized controlled trial using a 2 (gain vs. loss framing) × 2 (narrative vs. non-narrative framing) factorial design to implement the exercise rehabilitation education program, comprising four groups: Group A (gain-narrative framed message), Group B (loss-narrative framed message), Group C (gain-framed non-narrative message), and Group D (loss-framed non-narrative message). This phase includes a 3-week intervention period and a 3-month follow-up period. The study design is shown in Figure 1.

## Phase 1: development of an exercise rehabilitation education program for post-PCI patients based on message framing effect

### Qualitative study

A qualitative descriptive study will be conducted to gain insight into patients’ experiences, perceived barriers, expectations, and message needs, such as content preferences, access, and the form in which the message will be presented in relation to exercise rehabilitation after PCI. We will use a purposive maximum variance sampling strategy to recruit patients who have undergone PCI from the Department of Cardiology of a tertiary care hospital in Hangzhou, China. Participants must be adults (≥18 years old) who are clinically stable and assessed to be at low exercise risk after PCI, and all patients should sign an informed consent form. Patients with physical activity impairments, severe comorbidities, cognitive/communication impairments, or participation in other studies will be excluded. Qualitative data will be collected through semi-structured interviews using a protocol developed from a literature review and preliminary team discussions. Key topics include patient perceptions of exercise after PCI, past experiences, concerns, instruction received, and preferences for content and delivery. Interviews, lasting 30–45 min, will be audio-recorded, transcribed verbatim within 24 h, and supplemented by field notes. The transcribed texts will undergo independent coding by two researchers using thematic analysis with NVivo 12.0. Through an iterative process, codes will be compared, discussed, and refined to identify and agree upon key themes.

### Literature review and summary of evidence

A systematic evidence review will be conducted to ensure the scientific validity of the education program. A comprehensive literature search will be conducted in major electronic databases (including PubMed, Embase, Cochrane Library, CNKI and Wanfang) and guideline repositories (e.g., GIN, NICE, AHA and ESC). The inclusion criteria for the literature are as follows: (1) study population comprising post-percutaneous coronary intervention patients; (2) interventions involving cardiac exercise rehabilitation; (3) publication types limited to clinical guidelines, evidence summaries, recommended practices, expert consensuses, or systematic reviews. The exclusion criteria are as follows: (1) literature unrelated to the topic or duplicates; (2) conference abstracts, letters, incomplete data, or literature for which the full text is not available; (3) literature not published in Chinese or English. During the search process, a combination of subject terms and free terms will be used to search for core concepts.

### Delphi expert consultation

A Delphi expert correspondence method will be organized to refine and modify an initially developed exercise rehabilitation education program for patients after PCI. Experts from relevant fields such as cardiology, rehabilitation medicine, nursing, nursing education, and chronic disease management will be purposively selected. The criteria for selection of experts include: (1) undergraduate degree or higher; (2) intermediate or higher title; (3) more than 5 years of relevant work experience; (4) voluntary participation. The target number of experts is 15–50. The Delphi process will consist of two rounds of online consultations conducted via email or WeChat. In each round, experts will receive a questionnaire covering the following areas: their demographic and professional background, self-assessment of their judgment basis and familiarity with the content, and the draft education plan. They will be asked to rate the importance of each plan item on a five-point Likert scale and to provide open-ended revision comments. Data analysis, including assessments of expert response rates, authority coefficients, coordination of opinions, and concentration of feedback, will be performed using Excel 2019 and SPSS 25.0. We will organize discussions based on the feedback from experts to complete the revision and improvement. The screening criteria for items will be: a mean importance score >3.5 and a coefficient of variation <0.25. For those entries that do not meet the above criteria, the group will conduct a comprehensive discussion to collectively decide whether to retain, revise, or delete them.

### Pre-experimental

A purposive sampling method will be adopted to invite post-PCI patients hospitalized in the Department of Cardiology of a tertiary hospital in Zhejiang Province to experience the educational program. After obtaining the patients’ consent, they will be invited to watch the exercise rehabilitation education video and encouraged to ask any questions and give feedback, and the education program will be further modified and optimized according to the patients’ opinions.

### Final draft

Unlike traditional educational formats, this study will innovatively combine the theory of message framing effect, centring on the knowledge of exercise rehabilitation of aerobic exercise, resistance training and flexibility training, and present each exercise from the perspective of the benefits of exercise and the risks associated with inactivity. Real-life case studies will also be incorporated, resulting in the final draft of the rehabilitation education program for post-PCI patients. Through the combination of gain-loss framing and narrative framing, four groups of differentiated educational materials will be developed. The gain framing will emphasize the positive outcomes and benefits patients can achieve through exercise rehabilitation, such as improved cardiac function, reduced risk of cardiovascular events, enhanced physical strength, and improved quality of life. The loss framing will highlight the negative consequences and potential losses patients may face if they do not follow exercise rehabilitation recommendations. Additionally, by using specific patient stories of exercise rehabilitation and employing emotional narratives, the narrative framing will enhance patients’ emotional resonance and understanding, thereby better promoting the implementation of exercise rehabilitation education. Considering that most patients with coronary heart disease are older adults and that many experience age-related visual impairment, which makes reading and understanding text difficult, they tend to prefer receiving exercise rehabilitation education messages in video format for easier comprehension and learning. Based on the findings of the literature review and qualitative interviews, animated video presentations are therefore incorporated in addition to the text-based exercise rehabilitation education program. The videos will be designed to present the textual content of the educational messages in bold characters, with appropriate pictures, animations, voice-overs and background music to enhance the readability and interest of the videos, and the length of each video will be controlled to be within the range of 3–6 min.

## Phase 2: application of the exercise rehabilitation education program

### Study setting

This randomized controlled trial will use consecutive sampling to recruit participants from the cardiovascular department of a tertiary-level general hospital in Zhejiang Province.

### Patients

Patient recruitment will be managed by experienced clinical nurses. Before recruitment, these nurses will receive comprehensive training from the research team on the study’s objectives, methods, and procedures. Leveraging their familiarity with the patients’ specific conditions, the nurses will ensure a professional and efficient recruitment process. Following recruitment, a member of the research team will provide a standardized explanation of the study’s purpose and significance to all participants. Special emphasis will be placed on the confidentiality agreement, explicitly instructing patients not to discuss the intervention details with other participants. This process is designed to address patient concerns, enhance intervention adherence, and ensure the integrity of the trial. The researcher will assure participants that their personal information will remain strictly confidential and will be used solely for scientific research and publication. It will be clearly stated that participants can withdraw from the study at any time without penalty or consequence. Written informed consent will be obtained from all eligible participants before any data collection. Table 1 displays the patient inclusion and exclusion criteria, as well as shedding details.

**Table 1:** Patient inclusion, exclusion and shedding criteria.

Criteria	Content
Inclusion criteria	Patients who have undergone PCI; Patients aged greater than or equal to 18 years; Stable condition post-surgery, with no severe cardiovascular complications; Patients assessed as low-risk for exercise rehabilitation post-PCI according to the *Expert Consensus on Exercise Rehabilitation After Percutaneous Coronary Intervention;* Patients can manipulate a smartphone with WeChat independently, or have a co-residing primary caregiver (who fulfils these technical criteria) to assist them;
Exclusion criteria	Individuals with physical mobility impairments; Malignant tumors or other serious complications; Pregnant or breastfeeding individuals; Mental illness, cognitive impairments, or language communication barriers; Individuals currently participating in other studies;
Shedding criteria	Voluntary withdrawal from the study; Loss to follow-up; Participants failing to complete the video-based intervention as required; The occurrence of any serious adverse event or a significant deterioration in health status during the trial period;

### Sample size

The primary outcome indicator adopted in this study is exercise adherence. The sample size was calculated using the sample size estimation formula for multiple means in randomized controlled trials, accounting for a 20% dropout rate, resulting in a final sample size of 100 subjects. The sample size calculation was determined using PASS 2021 software.

### Randomization, allocation, and blinding mechanisms

This study employs a computer-generated simple randomization method. Before the trial, a researcher will use computer software to randomly generate a randomization number list, allocating patients to four groups in a 1:1:1:1 ratio: Group A (gain-narrative framed message), Group B (loss-narrative framed message), Group C (gain-framed non-narrative message), and Group D (loss-framed non-narrative message). The generated random number list will be stored in a sealed envelope until the randomization process is completed. The researcher responsible for generating the random sequence will not participate in the patient recruitment process.

This study will be blinded to the participants, who received the educational video via WeChat. During this process, patients will not be aware of which message-framed video they received. Meanwhile, the video interventions in the four groups will be designed to mask the content of the different screens through similar designs (such as textual descriptions, animations combined with case presentations), and the researchers will personalize reminders and instructions based on patients’ responses during the experiment, and will process the feedback through a standardized template to avoid disclosing the group characteristics and to ensure the effectiveness of the interventions. Unblinding may be performed during the trial when essential to ensure participant safety or to facilitate necessary therapeutic adjustments in the event of a serious adverse event to minimize any potential impact on study outcomes, as reported by the participant or as assessed by the study investigators and the treating physician. If unblinding is required during the trial, the reasons, timing, participant-related information, and the corresponding decision-making process will be fully documented and archived. All unblinding procedures will adhere to ethical standards.

### Interventions

The intervention content of this study will be based on the exercise rehabilitation education program. On the day of discharge, researchers will select a quiet private departmental meeting room and use a time-slot booking system to ensure that the four participant groups do not overlap. We will explain the purpose, methods and procedures of the intervention to patients face-to-face and implement the first intervention. Educational videos will be sent to participants via WeChat for viewing and learning, serving as the primary form of the intervention. Participants will be guided through the video-viewing process, and after viewing, they will be asked questions regarding the basic content presented in the video. If the patients are unable to recall the content, the video will be replayed until the patients indicate they understand the content. During the viewing period, any questions raised by the patients will be promptly addressed.

In weeks 1, 2 and 3 after discharge, the researcher will remind patients to watch the video after sending the corresponding study content and video every week. After viewing, participants will be invited to answer two brief questions related to the video content. The researcher will also maintain continuous communication with participants and will promptly address any questions raised during the study period. After completing each video study and test, patients will be praised and encouraged via WeChat to improve adherence. The four groups will receive different contents of short educational videos, but the mode, timing, and frequency of video delivery and question prompts are the same across groups. The health education content provided by medical staff during hospitalization and in routine post-discharge follow-up is also identical. In this study, scientifically validated exercise instruction videos will be provided, clearly listing contraindicated exercises and emergency stop signals. We will inform patients of the procedures to be followed in case of discomfort during exercise to ensure patient safety. The process of intervention implementation can be seen in Table 2.

**Table 2:** Process of implementation of the exercise rehabilitation education program.

Intervention time	Video theme	Frame				Intervention implementation
		Group A	Group B	Group C	Group D	
On the day of discharge	Post-PCI exercise rehabilitation education	Frameless	Frameless	Frameless	Frameless	Face-to-face, WeChat
1 week after discharge	Aerobic exercise guidance	Gain frame	Loss frame	Gain frame	Loss frame	WeChat
2 Weeks after discharge	Resistance exercise guidance	Gain frame	Loss frame	Gain frame	Loss frame	WeChat
3 Weeks after discharge	Flexibility exercise guidance	Gain-narrative frame	Loss-narrative frame	Gain frame	Loss frame	WeChat

During the intervention period, patients in all four groups can continue to receive their regular medical treatment and rehabilitation management (e.g., medication, dietary adjustments, psychological support, etc.), which are assessed to be compatible with the intervention content of this trial and are not expected to affect the effectiveness of the intervention. If patients need to receive other treatments (e.g., surgery, emergency treatment, etc.) or adjust their routine treatment due to health problems during the trial period, the research team will assess whether these treatments affect the efficacy and credibility of the findings of the trial and decide whether participants can continue to participate in the study. In addition, any additional treatments or medications other than routine treatment will be reported to the research team in advance and will be evaluated by the research team as appropriate to ensure that they do not adversely affect the trial intervention.

### Data collection and outcomes

Data will be collected at baseline before intervention, 1-month post-intervention, and 3-month post-intervention. At baseline, paper questionnaires will be distributed on the day of discharge before the first video viewing to collect data; at 1-month and 3-month follow-up, online questionnaires will be completed via Questionnaire Star. For those unable to complete the survey via Questionnaire Star, telephone follow-ups will be conducted. The questionnaires will be distributed and collected by trained researchers. The assessment tools used need to be fully validated and selected for their established reliability and validity in the original study, ensuring accuracy and comparability. In the process of completing the questionnaire, the researcher will answer the subjects’ questions on time, check for missing items after completing the questionnaire, and instruct the subjects to add a message as needed. After data collection is completed, the data will be entered promptly. Following data entry into the analysis system, independent double data checks will be performed by two researchers to ensure accuracy. This trial is a low-risk, non-pharmacological behavioral intervention; therefore, a formal DMC will not be established.

The assessment of general patient message, designed by the study team itself through literature review and group discussion, will be divided into 2 parts. Part 1 will be general demographic message, including gender, age, ethnicity, religious beliefs, literacy, marital status, mode of residence, occupation, monthly household income, and type of health insurance; part 2 will be disease-related information, including duration of coronary heart disease, current diagnosis, medication use and types of medication, comorbidities, and a history of acute myocardial infarction or cardiac surgery.

#### Primary outcome

The primary outcome indicator is patient exercise adherence. We will use the Exercise Adherence Scale developed by Weng (35) to assess patients’ exercise adherence. The scale contains 15 entries divided into three dimensions covering exercise adherence, exercise monitoring adherence, and actively seeking advice. The higher the total score of the scale, the better the patient’s exercise adherence.

#### Secondary outcomes

Secondary outcome indicators include willingness to exercise, fear of exercise, exercise self-efficacy and quality of life. The specific assessment tools are listed below:

Willingness to exercise: Willingness to exercise will be measured using the 3-item questionnaire used in the van‘t Riet et al. (36) study. Higher scores indicate a greater willingness to exercise in patients.
Exercise fear: Exercise fear will be measured by the Tampa Scale for Kinesiophobia Heart (TSK-Heart) (37), which consists of 15 items covering four dimensions: fear of injury, functional deterioration, exercise avoidance and perceived cardiac risk. The total score of the scale ranges from 15 to 60, with higher scores indicating greater exercise fear, and a total score of ≥37 being defined as high exercise fear.
Exercise self-efficacy: Exercise self-efficacy will be measured by the Cardiac Exercise Self-Efficacy Instrument (CESEI) (38), which consists of 16 items with a total score of 80. The higher the score, the greater the patient’s sense of cardiac exercise self-efficacy.
Quality of life: Quality of life will be measured using the Chinese cardiovascular quality of life questionnaire (CCQQ) for cardiovascular disease patients (39). The questionnaire is based on the definition of quality of life by the World Health Organization (WHO) and assesses the level of quality of life of patients from three aspects: physical, psychological and social. It is the first universal scale used for quality of life evaluation of patients with cardiovascular disease in China. The CCQQ scale consists of 6 dimensions, covering physical strength, medical condition, medical situation, daily situation, psychosocial and work situation, with 24 entries and a total score of 0–154 points. The scores are divided into the following levels: 0–68 indicates poor quality of life, 68–84 is average, 84–120 is moderate, and 120–154 is excellent quality of life. The questionnaire has been widely used in China to evaluate the quality of life of patients with cardiovascular diseases.

### Data statistics and analysis

Statistical analysis of the data will be performed using SPSS 25.0 and R 4.3.2 software. All statistical analyzes will be conducted using two-sided tests, with a significance level set at *p* < 0.05. Continuous variables will be described using mean ± standard deviation, while categorical variables will be expressed as frequency (n) and percentage (%). For intergroup comparisons of baseline data: continuous variables that follow a normal distribution will be compared using one-way analysis of variance (ANOVA); those that do not follow a normal distribution will be compared using the Kruskal–Wallis nonparametric test. Categorical variables will be compared using the chi-square test or Fisher’s exact test, as appropriate.

To comprehensively analyze changes in outcome indicators across different groups and time points following the intervention, data will be analyzed using a linear mixed-effects model (LMM). The gain-loss framing, narrative framing, and time will be included as fixed effects, while individual study participants will be included as random effects to account for individual variability. The LMM model will be constructed to assess the main effects of the gain-loss framing, narrative framing, and time, as well as their interaction effects. If the interaction effects are significant, further analysis of the simple effects of the message framing and time will be conducted to explore the impact of the intervention on outcome indicators at different time points. If interaction effects are not significant but main effects are, Tukey’s HSD *post hoc* comparison analysis will be further conducted to explore the independent effects of different message framing and time points on outcome indicators. The LMM model uses maximum likelihood estimation and is able to fit the model efficiently using all available data containing missing values, and is therefore robust to missing data.

## Discussion

### Limitations of the literature and strengths of the protocol

Existing literature on the relationship between message framing effects and health behavior has primarily focused on the role of single framing, neglecting the synergistic effects of different frame combinations. Additionally, there has been limited research on patients post-PCI, particularly regarding the application of narrative framing in exercise rehabilitation. The strength of this study lies in its pioneering integration of the gain-loss framing with the narrative framing, enabling a more comprehensive intervention strategy for patients after PCI through multi-dimensional assessment. Furthermore, the study employs a rigorous randomized and blinded design, minimizing bias and enhancing the internal validity of the results.

### Design justification and constraints

This study employs a 2 (gain vs. loss framing) × 2 (narrative vs. non-narrative framing) factorial design, which is relatively novel in the field of post-PCI patient exercise rehabilitation education. This design allows the researcher to test the independent effects of two or more factors at the same time, and more importantly, it can analyze whether there is an interaction effect between the different levels of these factors, i.e., whether the effect of one factor is dependent on the level of another factor. This means the design can not only obtain the message on the main effects of each factor, but also find out whether the factors act synergistically, antagonistically, or independently of each other, thus greatly improving the message output and experimental efficiency of the study, and avoiding the large amount of resources and time costs required to conduct single-factor experiments in multiple trials. It can further validate the unique value of the message framing effect in exercise rehabilitation education and provide new insights for future theoretical development in this field. The absence of a control group receiving conventional education means that we cannot directly determine if the message-framing interventions are superior to standard educational practices. To address this design limitation, this study strictly adopts a pre-post comparison format. Based on this, future studies may consider setting up a blank control group, where the control group receives routine exercise rehabilitation education without specific message framing interventions. By comparing the results of the intervention group with those of the blank control group, the effectiveness of the message framing intervention can be more accurately assessed, while eliminating other confounding factors, thereby enhancing the scientific rigor and reliability of the study conclusions.

As this is a single-center study conducted at a tertiary-level hospital in Zhejiang Province, the sample size is relatively small. This may result in certain regional limitations and insufficient sample representativeness, potentially limiting the generalisability of the study findings to patients in other regions or different types of hospitals. To enhance the external validity of the study results, future research could recruit more patients from multi-center, cross-regional studies, encompassing different types of healthcare institutions and social backgrounds, thereby providing a more comprehensive and accurate assessment of the impact of the message framing on post-PCI patient exercise rehabilitation.

During the implementation phase of the intervention, although we can remind patients to watch the video via WeChat or telephone, we still cannot guarantee that each patient has carefully watched all the video content, which may be a limitation.

### Implementation challenges and solutions

During implementation, the study faced major challenges, including high patient dropout rates and standardization of intervention content. To address these issues, the research team took the following measures: improving patient participation through WeChat red envelope incentives and regular follow-ups; developing standardized intervention videos and test questions to ensure consistency of content; and providing uniform training to researchers to reduce operational bias. In addition, the study further controlled for confounding factors through stratified Randomization and baseline data balancing.

## Ethics and dissemination

The study was approved by the Ethics Committee of the Second Affiliated Hospital, Zhejiang University School of Medicine, Hangzhou, China (Ethics approval: No. 2024-0576). The full trial protocol and statistical analysis plan are available in the Chinese Clinical Trial Registry. Before the investigation, the researchers will inform all participants about the purpose and procedures of the study, ensuring that their personal information will remain strictly confidential in accordance with data protection principles. The collected data can be used solely for scientific research and publication. Written informed consent will be obtained from all participants prior to data collection. Participants will be informed that their participation is entirely voluntary and that they may withdraw from the study at any time without penalty. Participants who self-report or are identified during follow-up as having sustained injuries or a deterioration in health directly related to the study procedures will have their participation in the relevant portion of the study terminated early, following physician evaluation. Affected individuals will receive appropriate medical treatment and corresponding compensation.

Any significant changes to the protocol will first be discussed within the research team, reviewed by the principal investigator, and subsequently submitted to the institutional ethics committee for approval. All relevant parties, including investigators and the trial registry, will be informed of the approved amendments.
